# Vascularised Brain Organoids: Engineering Strategies and Neurobiological Applications

**DOI:** 10.1111/cpr.70161

**Published:** 2026-01-11

**Authors:** Yeajin Song, Hyejin Jo, Seokchan Jeong, Inseon Kim, Seunghun S. Lee

**Affiliations:** ^1^ Department of Biomedical Engineering Dongguk University Seoul South Korea

**Keywords:** blood–brain barrier (BBB), neurovascular disease modelling, perfusion and microfluidic systems, regenerative medicine and cell therapy, vascularized brain organoids

## Abstract

Brain organoids have become an essential platform for studying human neural development and neurological disorders. Yet, one major limitation of conventional brain organoids is their lack of vascular structures. This deficiency restricts organoid size, contributes to necrotic core formation, and hampers their functional maturation. Introducing vascularization offers a compelling solution—it enhances nutrient delivery, supports neurogenesis, and fosters the development of interfaces that resemble the blood–brain barrier (BBB). In this review, we explore how vascularization enhances the structural and physiological relevance of brain organoids and its growing significance in disease modelling and therapeutic screening. We examine current methodologies for engineering vascularized brain organoids (vBOs), including co‐culturing with endothelial cells (ECs), transcriptional programming, tissue fusion techniques, microfluidic perfusion systems, and 3D bioprinting. These strategies vary in complexity, scalability, and the extent to which they achieve vascular integration. Functionally, vBOs demonstrate improved oxygen diffusion, enhanced synaptic development, and more robust barrier properties. Such advances enable modelling of complex neurovascular conditions like stroke, glioblastoma, and BBB dysfunction. Moreover, vBOs are emerging as valuable tools in developmental studies and personalised medicine, supporting patient‐derived modelling and large‐scale drug testing in BBB‐relevant contexts. Despite these advances, replicating the structural complexity, functionality, and long‐term stability of native vasculature remains challenging. We discuss current limitations and highlight innovative approaches, including the use of next‐generation biomaterials and dynamic perfusion technologies. Ultimately, vBOs mark a significant step towards creating physiologically accurate in vitro models of the human brain—offering new opportunities for neuroscience research, drug development, and regenerative medicine.

## Graphical abstract

This review highlights emerging strategies to engineer vascularized brain organoids—including endothelial co‐culture, genetic induction, perfusion systems, and in vivo transplantation—and their applications in modelling neurovascular diseases, evaluating BBB drug delivery, and advancing regenerative medicine.

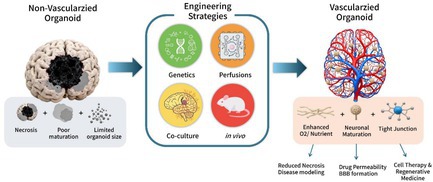

## 1 Introduction

As research on stem cells has advanced, their ability to differentiate into various cell types and self‐organise into tissue‐like structures has attracted significant attention. Based on these capabilities, scientists have successfully cultured stem cells in three‐dimensional environments to create organoids that closely resemble the structure and function of actual organs. In particular, studies using human induced pluripotent stem cells (hiPSCs) and embryonic stem cells (ESCs) [1, 2] have actively developed organoids of various organs such as the brain, pancreas, liver, and kidney [3, 4, 5, 6, 7, 8, 9, 10, 11, 12, 13, 14]. These organoids provide more physiologically relevant research models compared to traditional two‐dimensional cell cultures.

Brain organoids, three‐dimensional (3D) in vitro models derived from pluripotent stem cells, have significantly advanced our capacity to replicate early human brain development and study neurological diseases within physiologically relevant frameworks [15, 16]. These organoids spontaneously organise into layered structures that resemble the developing cortex and partially capture the diversity and architecture of brain cell types. Despite these advantages, conventional brain organoids face critical challenges, including necrotic core formation, restricted growth, and impaired functional maturation—issues largely attributed to the absence of vascular networks [17].

Emerging vascularization techniques offer promising avenues to address these shortcomings [18]. Various methods have been explored to enhance nutrient and oxygen delivery and promote maturation, such as co‐culturing with endothelial cells (ECs) [19, 20, 21, 22, 23], ectopic expression of vascular transcription factors like Ets Variant 2 (*ETV2*) [18, 22], transplantation into host animals [24, 25, 26], and integration with advanced microfluidic platforms [27, 28, 29]. However, each of these approaches presents its own limitations, such as incomplete BBB formation [30, 31], limited long‐term stability, or challenges in reproducibility. These challenges highlight the need for integrative strategies that combine multiple technologies—such as bioengineering, gene editing, and microfluidic systems—to overcome current constraints and further advance the vascularization of brain organoids [32, 33].

Despite significant progress in vascularization techniques, their practical utility and scalability vary across different engineering strategies. In vivo transplantation generates the most physiologically mature and perfused vasculature, yet its dependence on animal hosts, limited throughput, and batch variability restrict broad application in drug discovery or patient‐specific screening [34]. In contrast, microfluidic perfusion systems and 3D bioprinting–based perfusable scaffolds enable tightly controlled flow, shear stress, and long‐term tissue viability in fully in vitro environments, offering superior reproducibility and scalability. Recent developments in bioprinted vascular channels and modular organoid‐on‐chip platforms further enhance hierarchical vascular patterning and BBB‐relevant endothelial function, although they still fall short of the structural and dynamic complexity achieved in vivo [35, 36, 37, 38]. Accordingly, this review emphasises vascularization strategies that best balance physiological relevance with experimental scalability, including co‐culture–based approaches, genetic induction, and perfusion‐enabled platforms.

For disease‐focused applications, we highlight stroke, glioblastoma, and Alzheimer's disease as representative models because these disorders exhibit well‐defined neurovascular dysfunction—such as ischemic hypoxia, tumour‐driven angiogenesis, and BBB breakdown—that vBOs can robustly recapitulate. These diseases also provide quantifiable vascular and neuronal phenotypes suitable for benchmarking across emerging vBO platforms [39, 40, 41, 42]. In contrast, conditions such as vascular dementia or multiple sclerosis require systemic vascular risk factors, white‐matter perfusion deficits, or chronic adaptive immune contributions that remain difficult to reproduce reliably using current organoid systems. In particular, vascular dementia (VaD) depends on chronic small‐vessel insufficiency, progressive white‐matter ischemia, and long‐term cerebrovascular decline—processes deeply influenced by systemic cardiovascular conditions that remain beyond the scope of current vBO platforms [43, 44]. Multiple sclerosis (MS), likewise, is driven by cycles of adaptive immune activation, peripheral lymphocyte recruitment across the BBB, and chronic demyelination–remyelination dynamics—mechanisms that require mature neuroimmune interactions, myelinated white‐matter architecture, and functional immune infiltration, none of which are currently achievable in organoid models [45, 46]. For these reasons, VaD and MS were not included as primary disease examples, despite their significance as neurovascular disorders.

vBOs, owing to their enhanced physiological relevance, have emerged as versatile platforms for modelling neurovascular diseases [47], evaluating drug permeability, and advancing regenerative medicine [24]. By replicating key features of the neurovascular unit—including perfusable vessels and BBB‐like properties—these organoids enable more accurate simulations of pathological conditions such as stroke, Alzheimer's disease, and vascular dementia. Their ability to recapitulate ischemic or hypoxic environments facilitates mechanistic studies of neuronal injury and repair. In parallel, their use in BBB drug delivery testing allows for high‐throughput screening of CNS‐targeting compounds in a more predictive in vitro setting [48, 49, 50, 51, 52, 53]. Furthermore, vBOs show promise in cell therapy, where pre‐formed vascular networks improve graft survival and integration. When derived from patient‐specific iPSCs, they also support personalised modelling of neurovascular disorders and individualised therapeutic testing, particularly for genetically complex or rare conditions [54].

This review provides a comprehensive analysis of the diverse strategies currently employed to vascularize brain organoids, including co‐culture with ECs, genetic induction of vascularization, transplantation into host organisms, and integration with microfluidic systems. It further evaluates the functional advantages conferred by vascularization—such as enhanced neural maturation, blood–brain barrier (BBB) like properties and improved cellular viability and architecture—and highlights their applications in disease modelling, drug screening, and regenerative medicine. Additionally, this review critically examines the remaining technical challenges, including limited long‐term vascular stability and incomplete mimicry of the neurovascular niche [55, 56], and outlines future directions aimed at developing fully functional, physiologically relevant human brain organoid platforms through interdisciplinary approaches.

## 2 Importance and Functional Effects of Vascularization in Brain Organoids

The human brain is among the most metabolically demanding organs, relying on an intricate vascular network for continuous nutrient and oxygen delivery, waste clearance, and maintenance of the BBB. Conventional brain organoids, while revolutionary for modelling human neurodevelopment and disease, inherently lack vasculature. This absence results in hypoxia‐induced necrosis at the core, limited maturation of neural tissues, and a lack of physiologically relevant neurovascular interactions.

Incorporating vascularization into brain organoids offers a powerful solution to these challenges. Strategies such as co‐culture with ECs or genetic induction of vascular networks markedly enhance oxygen availability, cellular viability, and long‐term culture stability. vBOs exhibit improved neuronal maturation, spatial patterning, and synaptic functionality compared to their non‐vascularized counterparts. These changes effectively produce and secrete neurotransmitters, and neurons and glial cells are well arranged in a set location, such as the brain structure, which can greatly increase accuracy, reproducibility, and physiological similarity in disease modelling and drug screening.

Vascularization also plays a pivotal role in the partial reconstruction of the BBB—an essential interface that regulates molecular exchange and central nervous system (CNS) homeostasis. vBOs featuring perfused vessels and tight junction formations express key BBB‐associated proteins such as ZO‐1, occludin, and claudin‐5, and exhibit selective permeability akin to in vivo conditions. These features make vBOs highly valuable for CNS drug screening, offering more human‐relevant assessments of drug permeability and neurotoxicity.

Furthermore, the vascular niche actively regulates neural stem cell dynamics. ECs secrete signalling molecules like VEGF, BDNF, and Wnt, which influence neural progenitor proliferation, migration, and region‐specific differentiation. This enables the formation of distinct brain regions—such as cortex‐like or hippocampus‐like structures—enhancing the developmental fidelity of organoids.

The presence of vasculature also supports the metabolic coupling between neurons and glia. Through facilitated glucose and lactate transport, vBOs maintain prolonged neuronal activity and energy homeostasis. Additionally, improved perfusion contributes to electrophysiological maturation, evidenced by increased spontaneous firing, enhanced synaptic plasticity, and more robust neural network activity—features critical for modelling neurodegenerative and neurodevelopmental disorders.

Importantly, vascularization improves the structural and mechanical integrity of brain organoids. By promoting extracellular matrix (ECM) remodelling and tissue stiffness, vasculature enhances organoid resilience during extended cultures. Moreover, vascular elements provide instructive cues for self‐organisation and stem cell maintenance, contributing to more consistent organoid architecture [57, 58, 59]. vBOs also allow for the inclusion of immune cells such as microglia or peripheral leukocytes. This enables modelling of neuroimmune interactions relevant to Alzheimer's disease, multiple sclerosis, viral encephalitis, and brain tumours. In glioblastoma models, for example, vascularization enables the study of tumour angiogenesis, immune evasion, and drug penetration. Compared to animal models, vBOs offer distinct advantages in replicating human‐specific features of the BBB and immune responses. Their compatibility with patient‐derived hiPSCs also opens the door to personalised medicine applications, allowing researchers to evaluate individual‐specific drug responses and disease phenotypes in vitro.

In summary, vascularization is a critical advancement in organoid technology that addresses the limitations of conventional brain organoids while unlocking new opportunities for developmental biology, disease modelling, drug screening, and precision medicine.

## 3 Strategies to Achieve Vascularization in Brain Organoids

To provide an integrated overview of the major vascularization strategies discussed in this section, we first summarise key performance parameters—including technical complexity, scalability, and BBB‐related functionality—in a comparative table (Table 1). This framework offers readers a clear reference point before examining each strategy in greater detail.

**TABLE 1:** Quantitative comparison of major vascularization strategies based on complexity, scalability, and BBB integrity.

Vascularization strategy	Tecgnical complexity	Scalability	BBB integrity (Tight junctions, permeability)	Key characteristics
Co‐culutre with ECs	Low‐Medium	High	Low‐Medium	Simple and scalable Limited BBB maturation
Genetic Engineering (*ETV2, SOX17*)	Medium	Medium	Low‐Medium	Uniform EC induction Lacks perfusion
Perfusion/Microfluidic Systems	High	Low‐Medium	High	Strong BBB properties Dynamic flow
in vivo Transplantation	Very High	Very Low	Very High	Most mature vasculature Not scalable
Hybrid (Co‐culutre + Perfusion/Genetic + Transplantation)	Highest	Low‐Medium	Very High	Combines advantages of multiple strategies

### 3.1 Co‐Culture With Endothelial Cells

One of the most widely adopted strategies for introducing vascular features into brain organoids is co‐culture with ECs [19]. This approach involves incorporating ECs derived from primary, immortalised, or iPSCs into organoid cultures in the early or intermediate stages of development [18]. Generally, ECs and stem cells (embryocytes or induced pluripotent) can be mixed before the hBO protocol is initiated and induce spontaneous formation of vascular‐like structures. Importantly, the timing of endothelial cell integration plays a critical role, as early‐stage introduction may interfere with neural lineage specification, whereas late‐stage addition often results in poor vascular integration [60, 61].

Human umbilical vein endothelial cells (HUVECs) have been commonly used due to their accessibility and robust angiogenic ability [62]. They are collected from the endothelium of the umbilical cord vein. Alternatively, iPSC‐derived ECs are more physiologically relevant and provide a patient‐specific source [63, 64]. When cocultured under suitable conditions, these ECs can self‐organise into vascular‐like networks within cell organelles, sometimes forming light‐emitting structures and expressing EC markers such as CD31, VCAM‐1, and von Bilbranding factor (vWF). HUVECs can be cultured with mesenchymal stem cells (MSCs) derived from different origins. Adipose tissue mesenchymal stem cells (AD‐MSCs) and bone marrow mesenchymal stem cells (BM‐MSCs) are commonly known MSCs that are frequently co‐cultured with HUVECs for angiogenesis of various target tissues [65, 66, 67, 68, 69, 70]. BM‐MSC and AD‐MSC exhibit similar angiogenic abilities. However, the isolation process of AD‐MSC is more accessible than that of BM‐MSC [66, 67, 71, 72]. Recent approaches also explore the use of brain microvascular endothelial cells (BMECs), which exhibit more accurate BBB phenotypes, including tight junction formation and efflux transporter activity, thus offering improved physiological relevance compared to generic EC types like HUVECs [73].

Co‐culture promotes endothelial integration into the organoid matrix, promotes nutritional exchange, and supports neuronal survival [74, 75]. In some cases, ECs secrete trophic factors, which further affect neurogenesis and glial maturation [76]. Despite these advantages, the challenges still remain. Vascular networks formed by co‐culture are often not perfused and physiological fidelity is limited due to the lack of pericyte coverage [77] or adequate basement membranes [78, 79]. In addition, vascular formation occurs in self‐organising, resulting in heterogeneity between organoids that vary in vascular distribution or in each case of location and pattern. Due to these characteristics, spatial control over vascular tissue generally appears low. Moreover, although the presence of ECs may partly mimic aspects of the BBB, complete BBB function is still limited in these systems. For example, tight junction or selective permeability, which is a complete BBB property of the inner wall of blood vessels, is not sufficiently reproduced [23].

**FIGURE 1:**
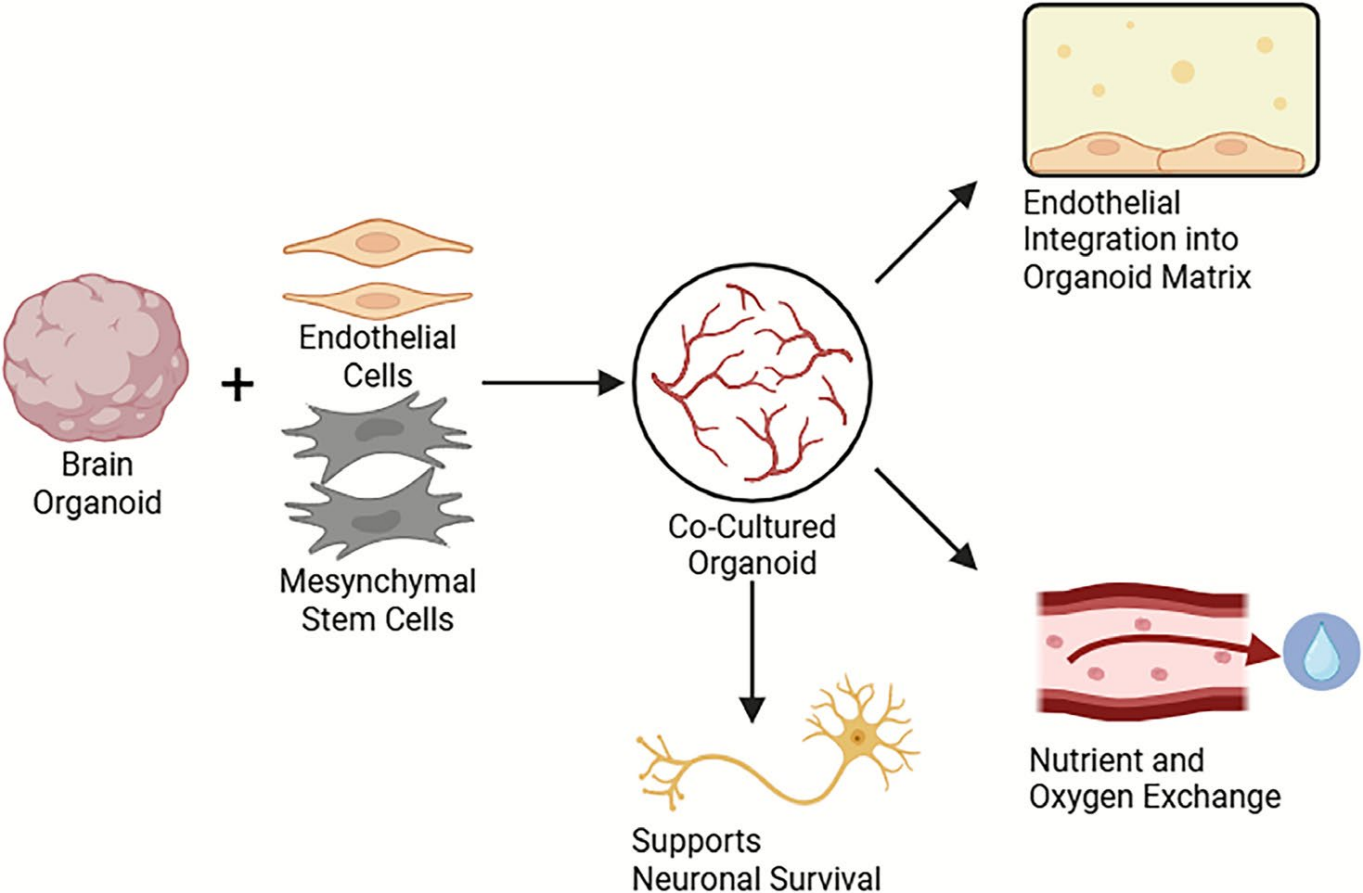
Co‐culture strategy for vascularization of brain organoids. Schematic representation of co‐culturing brain organoids with endothelial cells and mesenchymal stem cells to promote vascular integration. ECs and MSCs are introduced into developing brain organoids to support the formation of vascular‐like networks. This approach facilitates endothelial integration into the organoid matrix, enhances nutrient and oxygen exchange, and supports neuronal survival. Co‐culture with HUVECs or iPSC‐derived ECs is widely adopted due to its simplicity, scalability, and compatibility with standard organoid protocols.

Nevertheless, the coculture method introduces vascular urea into brain organoids, enabling direct interaction between brain cells and vascular cells. This not only facilitates improved nutrient and oxygen exchange but also supports neurovascular signalling, mimicking key aspects of the in vivo microenvironment. Due to its relative simplicity, cost‐effectiveness, and compatibility with existing organoid protocols, coculture remains the most accessible and scalable strategy. Moreover, it serves as a valuable foundational platform for integrating additional components—such as pericytes, astrocytes, or bioengineered scaffolds—to construct more physiologically accurate and functional brain models. As such, it lays essential groundwork for the development of next‐generation neurovascular organoid systems with enhanced complexity and translational relevance (Figure 1) [19, 20, 23].

### 3.2 Vascularization Genetic Engineering

Genetic engineering approaches have been used to induce endogenous vascularization within brain organoids without the need for external endothelial cell co‐culture [79]. This strategy typically involves ectopic expression of key transcription factors or signalling molecules that promote endothelial fate, such as *ETV2*, a master regulator of vascular development [80, 81]. Recent studies suggest that combining *ETV2* with other vascular regulators such as SRY‐box transcription factor 17 (*SOX17*) or Vascular endothelial growth factor A (*VEGF‐A*) can enhance endothelial maturation and functional integration, mimicking more closely the stepwise process of vascularization [22, 82]. For example, Cakir et al. introduced an inducible *ETV2* expression system in human pluripotent stem cells (hPSCs), enabling them to generate vBOs with internally derived endothelial‐like cells [18]. These engineered organoids have developed complex vascular‐like networks expressing canonical endothelial markers, including CD31, VE‐cadherin, and vWF, and in some cases have formed lumenized structures.

Compared with the co‐culture approach, genetically induced vascularization may result in a more uniform vascular distribution throughout the organoid, as vascular progenitors are generated endogenously in the early developmental stages [83]. This method also allows spatiotemporal control of vascular induction via an inducible promoter or CRISPR activation system. Despite the uniform induction of endothelial identity, spatial patterning of vascular architecture remains poorly controlled, often resulting in non‐physiological or randomly distributed vessel networks. Although real blood vessels are precisely regulated for guidance, branching, and pruning, gene‐induced vascularization lacks such spatial structure formation.

However, this approach has limitations. During *ETV2* induction, cells with endothelial cell characteristics are arranged like blood vessels to form a tubular structure, but no blood flows within this structure. As a result, the functions of real blood vessels, such as oxygen, nutrient supply, and waste removal, cannot be performed. As a result, vessels produced by *ETV2* overexpression often lack a response to the fully functional properties of mature ECs, such as selective permeability, tight junction formation, and response to shear stress. In addition, in static culture systems, the absence of blood flow or perfusion pressure interferes with the maturation and stabilisation of engineered blood vessels [84, 85].

To further increase the technical robustness of *ETV2*‐based strategies, it will be important to systematically quantify both endothelial differentiation efficiency and vascular function in these organoids. Recent studies demonstrate that *ETV2* overexpression can yield highly efficient endothelial differentiation when appropriately timed or modulated, achieving 89%–95% EC conversion across multiple pluripotent stem cell lines using modified mRNA delivery [86] and up to 99% CD31+ CD144+ purity within 5 days using optimised transcriptional activation protocols. Similar results have been observed in somatic stem cell systems, where *ETV2* upregulation significantly enhances endothelial lineage commitment and promotes robust tubulogenesis [87]. Despite these advances, most *ETV2*‐induced vBO studies still rely largely on marker expression rather than quantitative metrics such as network coverage, EC purity, or lumen formation efficiency. Functional vascular assays widely used in conventional endothelial and organ‐on‐a‐chip platforms—including tube‐formation analysis, transendothelial electrical resistance (TEER) measurements, and fluorescent tracer permeability tests—remain rarely applied in *ETV2*‐engineered organoids. However, established EC and BBB model literature emphasises the importance of these assays: Permeability and tube‐formation testing are recognised as critical benchmarks for validating EC maturity, while TEER and tracer permeability provide standardised, quantitative measures of BBB‐like barrier integrity [88]. Incorporating these functional readouts into future vBO studies would substantially strengthen the comparability, physiological fidelity, and translational relevance of genetically programmed vascular networks.

Another important yet underexplored aspect of engineered vascular networks is arterial–venous specification. Current *ETV2* or transcription factor–based vascularization approaches primarily generate generic endothelial tubes without clear segregation into arterial, venous, or capillary‐like identities. However, arterial–venous fate is governed by well‐established developmental signalling pathways: Notch–Delta‐like ligand 4 (DLL4) activation is essential for arterial identity and drives markers such as EphrinB2 and Neuropilin‐1 [89, 90], whereas COUP‐TFII suppresses arterial programs—including Dll4 and Hey1/2—to maintain venous identity [90]. Molecular profiling studies further reveal distinct transcriptional signatures that differentiate arterial, venous, and capillary ECs during development [91]. These pathways are also responsive to biomechanical cues. For example, shear‐stress regimes can bias EC fate towards arterial‐like or venous‐like states depending on magnitude and pattern, and engineered microenvironments combining biomaterials with controlled flow have been shown to induce AV‐specific phenotypes in vitro [92]. The absence of such subtype‐specific cues in current vBOs may limit their ability to recapitulate physiological hemodynamics, shear‐stress gradients, or region‐specific vulnerability to hypoxic or inflammatory injury [93]. Integrating transcription factor programming with biomechanical stimulation, AV‐specific reporters, or microfluidic flow systems therefore represents a promising route towards reconstructing hierarchical vascular trees within brain organoids [94].

**FIGURE 2:**
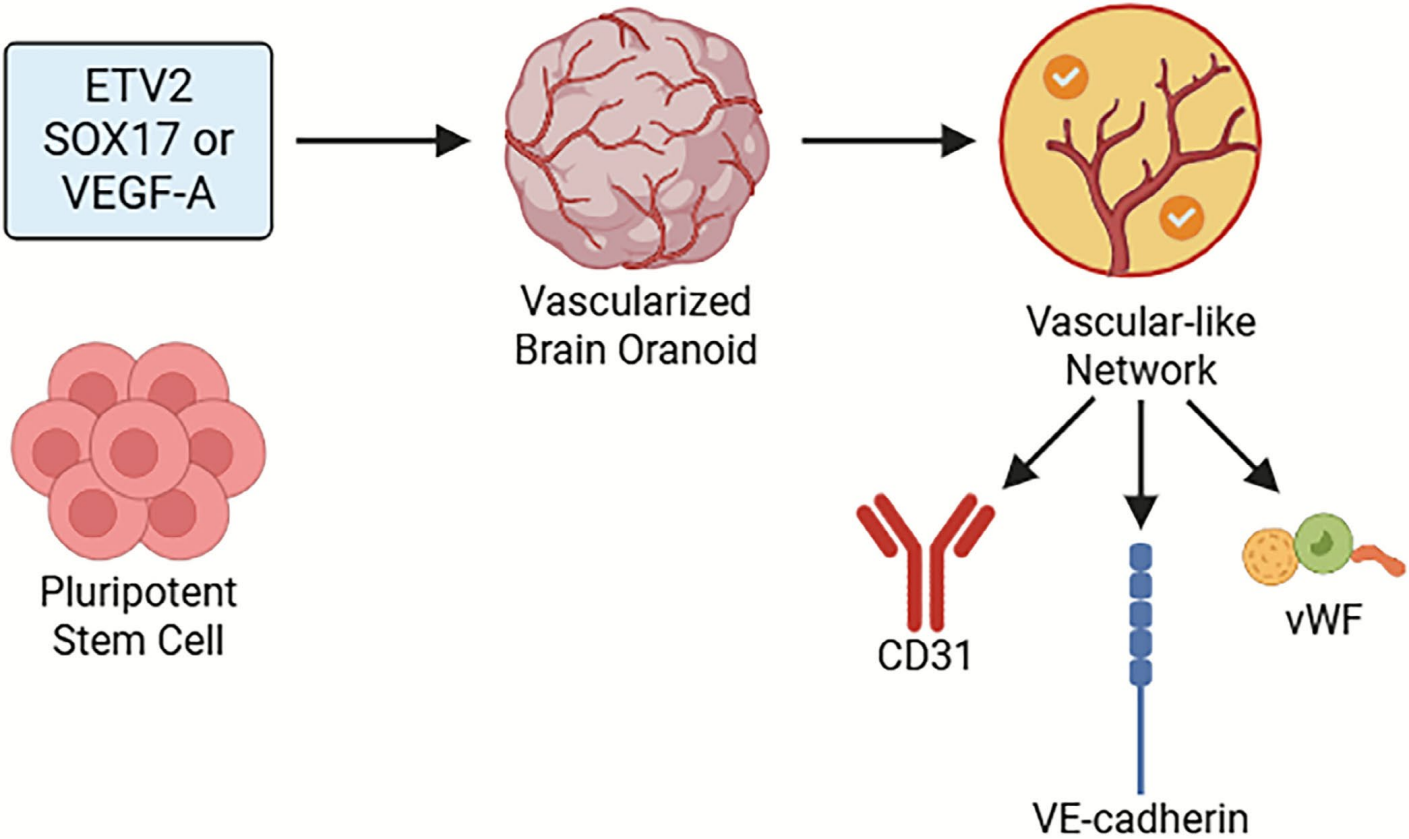
Genetic engineering strategy for vascularization of brain organoids. Schematic illustration of genetically induced vascularization via *ETV2*‐mediated endothelial differentiation. Pluripotent stem cells are engineered to express key transcription factors such as *ETV2*, *SOX17*, or *VEGF‐A*, inducing endogenous endothelial differentiation during organoid development. This leads to the formation of vascular‐like networks expressing canonical endothelial markers including CD31, VE‐cadherin, and von vWF. Compared to co‐culture approaches, this method promotes more uniform vascular integration within brain organoids, though it lacks physiological perfusion and spatial patterning of real vasculature.

Despite these drawbacks, genetic engineering provides a powerful platform for integrated vascularization, especially when combined with microfluidic perfusion systems or in vivo transplantation [95]. Future advances in synthetic biology and gene editing may further improve the accuracy and efficacy of this strategy. Moreover, while *ETV2*‐mediated induction shows great promise in generating vascularized organoids with internal consistency, it lacks precise spatial patterning and functional perfusion. To overcome these shortcomings, future efforts may focus on combinatorial gene activation (e.g., *ETV2* with *VEGF‐A* or *SOX17*), integration with microfluidic flow systems, or guided tissue architecture through bioprinting or ECM engineering. Additionally, regulatory and biosafety aspects of gene‐edited organoids must be carefully considered for clinical translation (Figure 2) [80, 82, 96].

### 3.3 Perfusion‐Based Vascularization

Perfusion‐based strategies aim to overcome the inherent diffusion limitations of oxygen, nutrients, and metabolic waste in brain organoids by mimicking vascular networks through the integration of microfluidic flow systems or engineered perfusable channels. These strategies facilitate the exchange of soluble factors deep within the organoid core, thereby reducing hypoxic and necrotic regions that typically develop in non‐vascularized, static cultures [94]. Unlike co‐culture or genetic engineering approaches, which mainly focus on cellular‐level vascular mimicry, perfusion‐based methods provide physiologically relevant biomechanical cues—most notably shear stress—that are essential for the differentiation, polarization, and long‐term functionality of ECs. Shear stress not only influences endothelial alignment and tight junction formation but also triggers gene expression patterns associated with vascular stability and BBB integrity [97]. By reproducing these dynamic physical stimuli, perfusion‐based systems more closely approximate the in vivo vascular microenvironment [98].

The microfluidic device is a mechanical device in which fine channels are densely formed, allowing precise control of the perfusion. In one study, microfluidic devices are integrated with brain organoids to mimic blood perfusion, enabling continuous media flow. It allows the medium with oxygen and nutrients to continuously flow through the microchannels on both sides, acting like the bloodstream. These systems support long‐term cultures by maintaining oxygen gradients and facilitating waste elimination, thereby facilitating the growth of larger, physiologically relevant organoids. For example, Wang et al. engineered a perfusion BBB chip connected to a brain organoid chamber, improving barrier integrity and endothelial morphology [78, 99].

Another strategy is to create hollow blood vessels by bioprinting the perfused channels or by inserting sacrificial hydrogels that can be removed later [78]. These artificial channels can be lined with ECs or coated with ECM to enhance compatibility and enable vascular infiltration. Combined with brain organoids, these systems have been shown to have a smooth supply of oxygen and nutrients, resulting in enhanced cell viability, reduced hypoxia, and more organized neurodifferentiation. This results in more complex and realistic neural networks [100].

**FIGURE 3:**
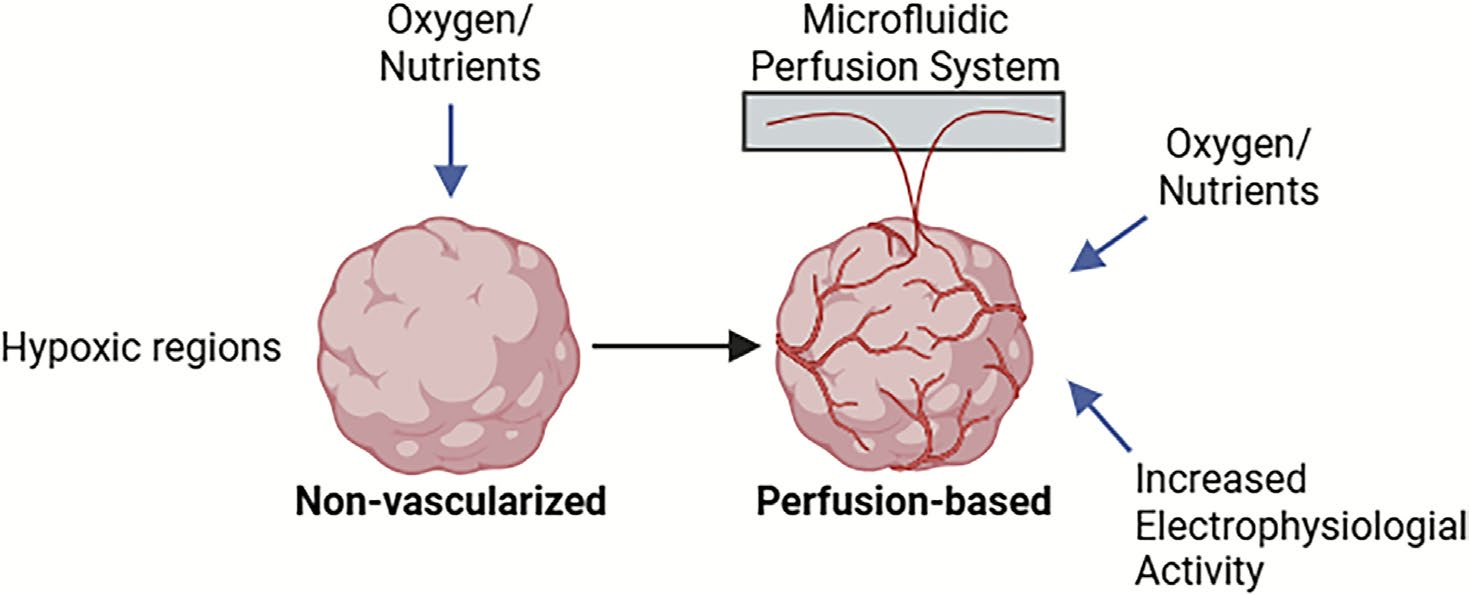
Importance of vascularization through perfusion in brain organoids. Comparison between non‐vascularized and perfusion‐based brain organoids. Non‐vBOs often develop hypoxic regions and necrotic cores due to limited diffusion of oxygen and nutrients. In contrast, perfusion‐based systems incorporating microfluidic channels enhance oxygen and nutrient delivery, reduce necrosis, and support functional neuronal maturation. These systems provide shear stress and other mechanical cues that promote endothelial differentiation and electrophysiological activity, closely mimicking the in vivo vascular environment.

Perfusion‐based methods also enable real‐time monitoring and access to the internal environment of organoids for drug delivery research or disease modelling [101]. However, the implementation of these methods is technically complex, requiring precise control over specialised equipment and flow dynamics. Nevertheless, perfusion‐based strategies not only mitigate diffusion limits but also provide mechanical stimuli critical for vascular maturation. By enabling shear stress and convective transport, they prevent hypoxic core formation and enhance endothelial and neural development. These systems also offer real‐time monitoring and precise environmental control, making them promising platforms for disease modelling and drug screening despite their technical demands (Figure 3) [18, 102].

### 3.4 In Vivo Transplantation‐Based Vascularization

In vivo transplantation provides a powerful strategy to utilise the natural blood vessels of the host to achieve functional vascularization of brain organoids. When transplanted into immunodeficient mice, brain organoids can be quickly vascularized by host‐derived ECs, which can penetrate the organoids to form lumenized blood vessels. These blood vessels are connected to the systemic circulation, providing essential oxygen and nutrients, improving the organoids' survival and growth. Immunodeficient mouse models, such as non‐obese diabetes‐severe combined immunodeficiency (NOD‐SCID) IL‐2Rγ null (NSG) mice, are used to prevent xenograft rejection and facilitate long‐term engraftment of human organoids without immune‐mediated interference. In vivo transplantation not only provides vascular integration but also exposes the organoids to other host‐derived physiological cues such as inflammatory signals, mechanical forces, and neural activity, which can further influence neurodevelopment, gliogenesis, and circuit formation. Moreover, host‐derived ECs that infiltrate the organoid can begin to express tight junction proteins and other markers associated with the BBB, offering a unique platform to investigate human BBB development and function in vivo [25].

This approach was first demonstrated by Mansour et al. implanted human brain organoids into the mouse cortex and observed host‐derived CD31^+^ vessels penetrating deep into the organoid structure within a few weeks. The vascularized organoids showed decreased necrosis, improved neuronal maturation, and axon growth compared to the non‐transplanted controls. Functional integration of host vasculature has been validated by perfusing fluorescent dextran or oxygen‐sensitive dyes, confirming that the newly formed vessels within the organoids are capable of active blood flow and oxygen delivery [26].

**FIGURE 4:**
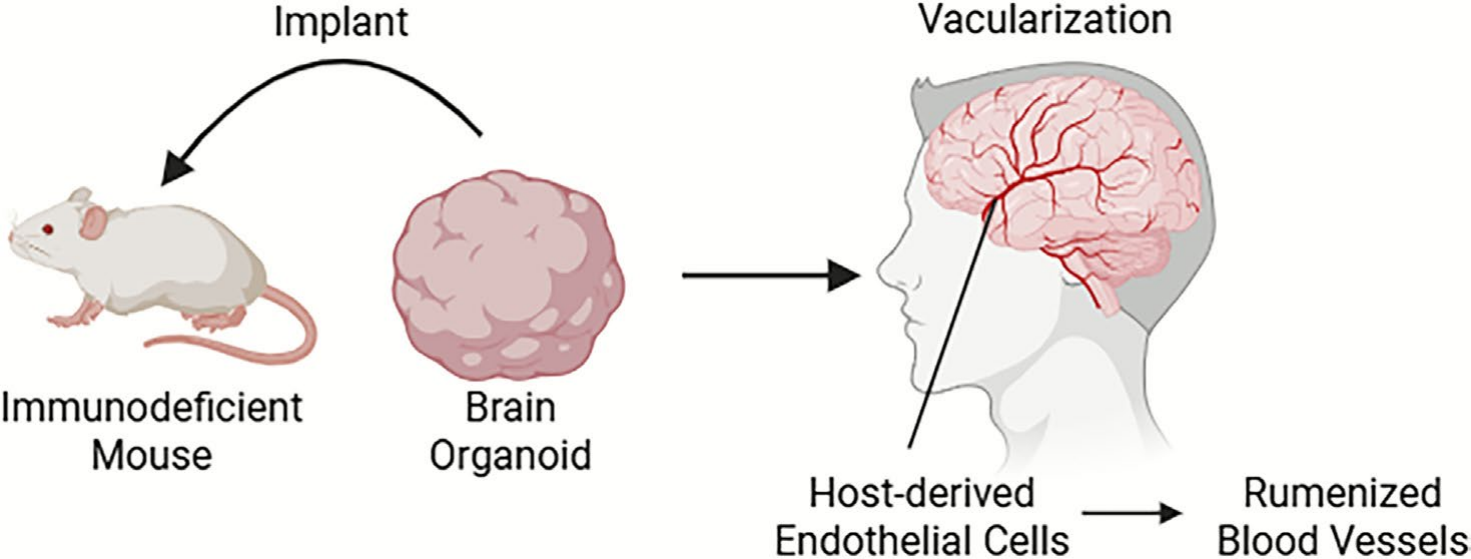
In vivo transplantation enables host‐mediated vascularization of brain organoids. Overview of in vivo transplantation‐based vascularization of brain organoids using immunodeficient mouse models. Human brain organoids transplanted into the brains of immunodeficient mice become vascularized by host‐derived ECs, which infiltrate the organoids and form functional, lumenized blood vessels connected to the host circulatory system. This vascular integration improves nutrient and oxygen delivery, supports organoid survival, and promotes neuronal maturation. The model also enables the study of BBB development and host‐organ interaction in a physiologically relevant context.

In addition to passive integration, co‐transplantation of organoids with ECs or MSCs has been shown to enhance vascular development and support neurogenesis [103]. In addition, transplantation to areas with high blood vessels, such as subarachnoid space or striatum, can improve the survival and integration of grafts. Importantly, in vivo models offer opportunities to study vascular‐organ interactions, BBB formation, and long‐range neural projections in physiologically relevant environments. Despite these advantages, species differences between human organoid cells and murine host vasculature can introduce variability in molecular signalling, potentially affecting the fidelity of human‐specific developmental processes. Moreover, this method poses ethical issues, limits access due to animal model requirements, and challenges high‐throughput screening or patient‐specific research. Recent advances such as cranial window techniques and intravital imaging allow for real‐time monitoring of transplanted organoids, enabling dynamic assessment of vascular integration, neuronal activity, and disease progression over time (Figure 4) [75].

### 3.5 Hybrid Approaches

Hybrid strategies aim to combine the strengths of multiple vascularization techniques to overcome the limitations inherent in individual approaches. These methods incorporate aspects of coculture, genetic engineering, perfusion, and in vivo transplantation to create more mature, functional, and scalable vBOs.

**FIGURE 5:**
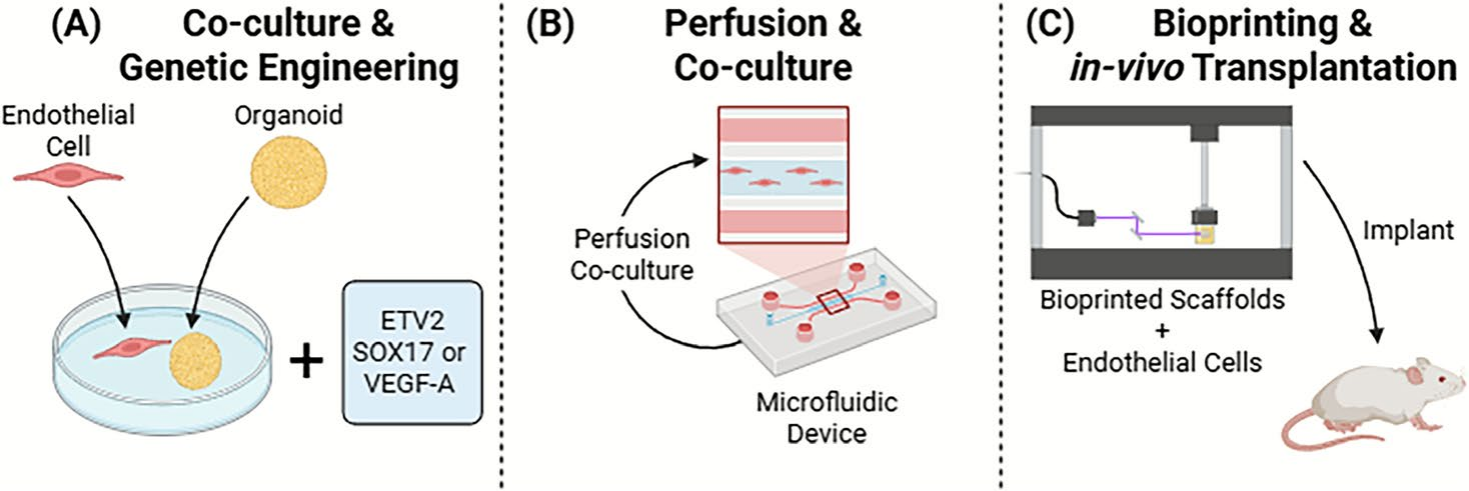
Hybrid strategies combining multiple vascularization approaches in brain organoids. Illustration of hybrid vascularization approaches integrating co‐culture, genetic engineering, perfusion systems, bioprinting, and in vivo transplantation. (A) Co‐culture & Genetic Engineering: ECs are co‐cultured with organoids, while transcription factors such as *ETV2*, *SOX17*, or *VEGF‐A* are genetically introduced to pluripotent stem cells to enhance endothelial differentiation. This dual strategy promotes spontaneous vascular network formation within organoids and improves endothelial identity and marker expression (e.g., CD31, VE‐cadherin). (B) Perfusion & Co‐culture: Microfluidic devices enable dynamic perfusion to mimic blood flow. Combined with co‐cultured ECs, this system introduces shear stress and improves mass transport (oxygen, nutrients), thereby enhancing endothelial barrier function and electrophysiological maturation of the organoids. These platforms allow for real‐time monitoring and high‐throughput drug screening applications. (C) Bioprinting & in vivo Transplantation: Bioprinted scaffolds are seeded with ECs and implanted into immunodeficient mouse brains. This enables rapid vascular integration with host vasculature, supporting perfusion, nutrient delivery, and tissue remodelling. The spatially controlled vascular structures support complex neurovascular architecture and provide an in vivo‐like environment.

For example, Cakir et al. combined endothelial programming induced by *ETV2* with in vivo transplantation to enhance vascular integration and maturation in brain organoids. *ETV2*‐expressing cells formed vascular‐like networks in vitro, which, upon transplantation, are linked to host blood vessels to improve oxygen diffusion and support neurodevelopment. In addition, oxygen supply and waste removal are possible as blood flows, compensating for the effects that cannot be obtained with *ETV2* gene manipulation alone (Figure 5A) [18].

Other approaches integrate dynamic perfusion and coculture systems. Microfluidic perfusion systems have been used in conjunction with cocultured endothelial and perivascular cells to enable simultaneous dynamic flow and intercellular interaction. These systems more accurately recapitulate BBB properties and enable real‐time drug testing than static cultures. In the study of the high‐throughput microvascular co‐culture model using the PREDICT96 platform, a co‐culture model was constructed by culturing human retinal microvascular ECs and pericyte on both sides of a microporous membrane. The system contains 96 bilayer microfluidic devices, each of which can independently control fluid flow, allowing for simultaneous assessment of various experimental conditions. The co‐culture has improved barrier function over monoculture and increased responsiveness to inflammatory stimulation and fluid shear stress (Figure 5B) [104].

Some researchers employ bioprinted scaffolds as a hybrid strategy. These scaffolds, inoculated with genetically modified or pre‐differentiated ECs, can be implanted in vivo to facilitate rapid anastomosis with host blood vessels. Such approaches leverage the natural remodelling capabilities of the host system while providing spatially controlled vascular templates. In one study, ECs and fibroblasts were inoculated into collagen scaffolds to induce pre‐vascularization. Transplantation of these scaffolds in vivo revealed rapid integration and vascularization with host tissues, which have been suggested as useful strategies for tissue engineering applications (Figure 5C) [71]. A major advantage of hybrid strategies is the inclusion of multiple supporting cell types—including pericytes, astrocytes, microglia, and immune cells—which more faithfully replicate the complex neurovascular microenvironment. These cells play essential roles in vascular stability, BBB integrity, and neural tissue homeostasis, enabling more physiologically relevant intercellular communication [25]. By combining diverse vascularization methods, hybrid systems promote long‐term vascular stability and maturation, improving oxygen and nutrient delivery as well as vascular signalling necessary for neurogenesis, gliogenesis, and neural circuit formation. The spatial and temporal precision offered by genetic engineering, combined with mechanical stimuli from perfusion and physiological conditions of in vivo transplantation, addresses key limitations of single techniques [18, 22]. Hybrid models also show great potential for precision medicine. Integration of patient‐derived stem cells and gene editing allows for generation of personalised brain organoids, facilitating disease modelling for neurological disorders such as Alzheimer's disease, stroke, and brain tumours. These organoids offer platforms for targeted therapeutic screening and better representation of patient‐specific phenotypes [105, 106]. Nevertheless, hybrid strategies are technically complex, requiring interdisciplinary expertise in stem cell biology, tissue engineering, microfluidics, and animal surgery. Ethical considerations, especially regarding human‐animal chimeras, must also be carefully navigated. In addition, standardisation and scalability remain significant hurdles to clinical and pharmaceutical translation. Looking ahead, future integration with emerging technologies—such as AI‐driven culture optimization, advanced 3D bioprinting using biomimetic materials, and real‐time biosensor monitoring—may further refine hybrid strategies. These advancements could enable high‐throughput, reproducible production of functionally vBOs, revolutionising in vitro modelling of human brain development, neurovascular diseases, and drug delivery platforms. Hybrid systems leverage the complementary strengths of each approach, promoting vascular stability, BBB formation, and neuronal development. They offer promising platforms for disease modelling, personalised medicine, and translational neurovascular research.

The vascularization strategies are not interchangeable tools but platforms with distinct strengths that align with specific experimental goals [29, 107]. For applications that demand high‐throughput, quantitative readouts—particularly BBB permeability assays and CNS drug screening—perfusion‐based microfluidic systems are especially powerful [107]. By enabling controlled flow and continuous medium exchange, these devices support TEER measurements and real‐time assessment of tight junction integrity and transporter activity, such as P‐glycoprotein (P‐gp) and glucose transporter 1 (GLUT1), under defined shear stress conditions. Recent organoid‐on‐chip studies have shown that perfused vBOs exhibit more mature BBB‐like phenotypes, reduced hypoxia, and transcriptomic profiles that more closely resemble in vivo vasculature than organoids maintained under static culture. In contrast, in vivo transplantation‐based vascularization is best suited for questions that require physiological perfusion, systemic signalling, and evaluation of long‐term graft integration—conditions that are critical for cell therapy and regenerative medicine. Following engraftment into immunodeficient rodent brains or extraembryonic membranes, host‐derived vessels rapidly invade and anastomose with the organoid vasculature, leading to lumenized, blood‐perfused networks, increased neuronal maturation, and reduced apoptosis. Such models uniquely allow investigators to monitor functional recovery, neuroinflammation, and host–graft interactions over extended periods, thereby providing an essential bridge towards translational applications. Co‐culture and genetic engineering approaches occupy an intermediate space: they are relatively accessible and scalable, making them well suited for mechanistic studies of neurovascular interactions, early disease phenotyping, and pilot drug testing [108]. Coculture of brain organoids with HUVECs or brain microvascular ECs can reduce hypoxia and necrosis while promoting neurogenesis and partial BBB‐like features, including tight junction marker expression and improved barrier selectivity [108, 109]. Transcription factor–based programming (e.g., *ETV2, SOX17*, or *VEGF‐A*) further enables spatiotemporal control over endothelial induction and more homogeneous vascular distribution within organoids. Building on these advances, hybrid systems deliberately combine multiple strategies—for example, genetically vascularized organoids integrated into perfusion chips or pre‐vascularized assembloids subsequently transplanted in vivo—to leverage complementary benefits of each approach and support complex, multi‐scale disease modelling and personalised medicine [29, 107, 109].

Taken together, matching the vascularization method to the intended application is key: perfusion‐based systems are ideal for BBB‐relevant drug screening, in vivo transplantation is indispensable for cell therapy and regenerative studies, co‐culture and genetic engineering facilitate developmental and mechanistic investigations, and hybrid strategies are emerging as versatile platforms for precision neurovascular research [109]. In the following sections, we discuss how these distinct yet complementary strategies are being deployed for neurological disease modelling, drug permeability and BBB testing, and regenerative medicine using vascularized brain organoids.

## 4 Application of Vascularized Brain Organoids

**FIGURE 6:**
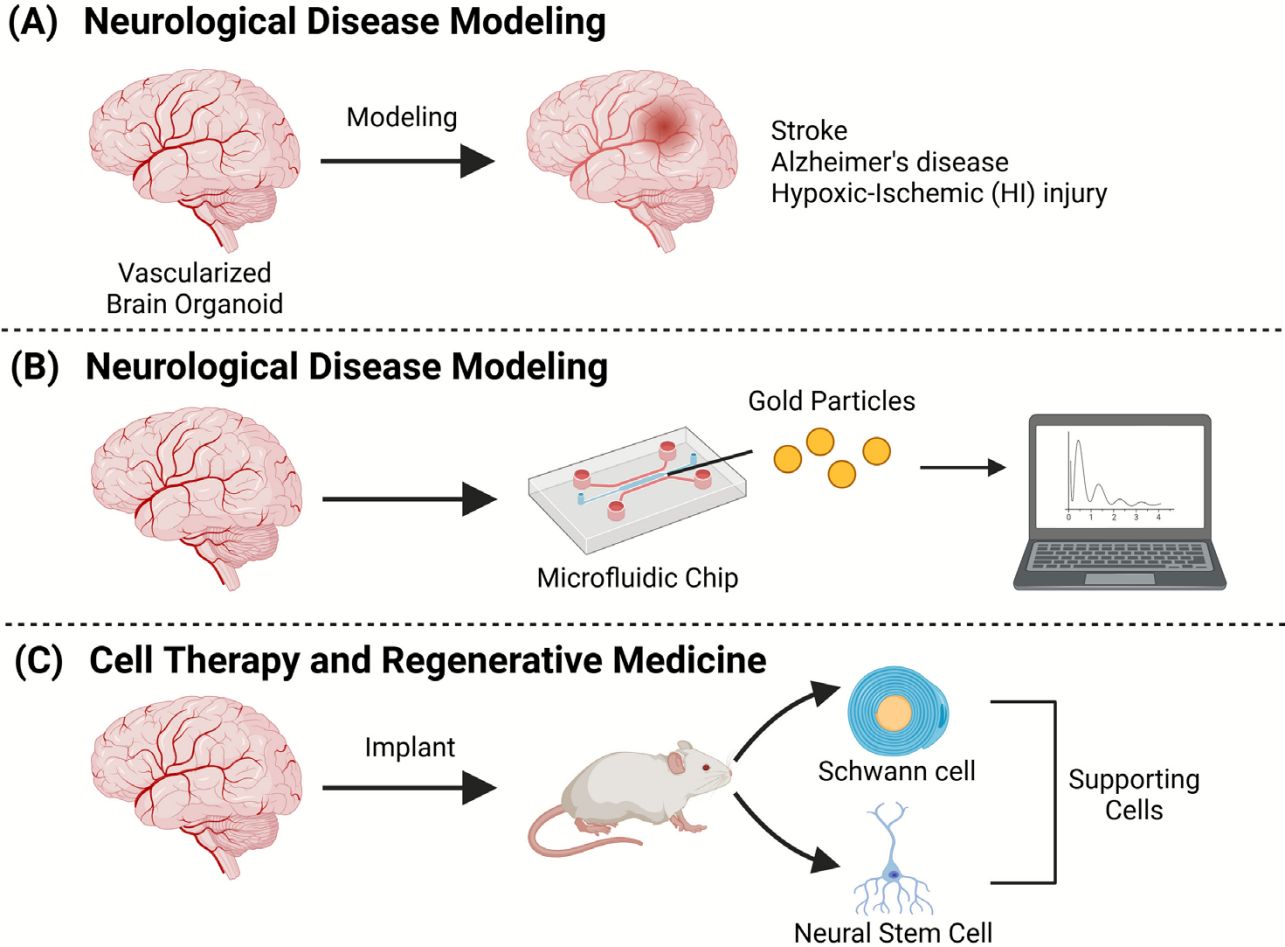
Applications of vascularized brain organoids in disease modelling, drug screening, and regenerative medicine. Schematic illustration of the three major applications of vBOs. (A) Neurological Disease Modelling: VBOs serve as in vitro platforms to model complex neurovascular disorders such as stroke, Alzheimer's disease, and hypoxic–ischemic (HI) injury. The integration of vascular structures enables simulation of hypoxic conditions and supports mechanistic studies of neuronal injury, inflammation, and disease‐specific pathology. (B) Drug Screening and Blood–Brain Barrier (BBB) Permeability Testing: These organoids replicate key BBB characteristics, including tight junctions and drug efflux proteins (e.g., P‐glycoprotein [P‐gp]). When combined with microfluidic chips, they enable real‐time monitoring of compound transport, efflux activity, and nanomedicine permeability (e.g., gold nanoparticles), offering a reliable model for CNS drug delivery evaluation. (C) Cell Therapy and Regenerative Medicine: Pre‐vascularized cerebral organoids enhance graft survival and promote integration with host vasculature upon transplantation into animal models. Co‐transplantation with engineered neural stem cells or supportive cell types (e.g., Schwann cells, OECs) further supports tissue remodelling and functional recovery. Autologous iPSC‐derived organoids also offer immune‐compatible, patient‐specific therapeutic options.

Recently, brain organoids have emerged as valuable preclinical tools for modelling human brain development and disease mechanisms, and they are widely used in disease modelling and drug screening. However, conventional non‐vascularized organoids face limitations in oxygen and nutrient supply, which restrict long‐term culture and the replication of complex physiological responses. To overcome these challenges, vBOs have been developed by incorporating vascular structures such as ECs. These advancements improve the viability and maturation of organoids and enable more precise exploration of neurological disease pathophysiology, BBB properties, and regenerative therapeutic potential. This section discusses the applications of vBOs in three major areas: neurological disease modelling, drug permeability testing, and regenerative medicine and cell therapy (Figure 6).

### 4.1 Neurological Disease Modelling

vBOs offer a powerful platform to model complex neurological diseases associated with neurovascular dysfunction such as stroke, Alzheimer's disease, and vascular dementia. The incorporation of vascular networks enables simulation of hypoxic or ischemic states, facilitating mechanistic studies on neuronal injury, angiogenesis, and post‐ischemic repair. To contextualise these advantages in a more quantitative manner, we provide a summary table comparing the general functional performance of vascularized and non‐vascularized organoids across key physiological domains (Table 2). These functional differences form the foundation for understanding how vascularized organoids reproduce disease‐specific pathologies more faithfully. Consistent with these improvements, recent studies have applied vascularized cerebral organoids to model hypoxic–ischemic injury, stroke, and Alzheimer's disease with enhanced pathological fidelity.

**TABLE 2:** Summary of functional performance improvements in vascularized brain organoids compared to non‐vascularized organoids.

Functional domain	Non‐vascularized organoids	Vascularized organoids (vBOs)	Improvement level
Oxygen and nutrient diffusion	Hypoxic/necrotic core formation	Reduced hypoxia and minimal necrosis	Very High
Neuronal maturation	Immature electrophysiology, weak synaptic activity	Enhanced firing, strengthened synaptic plasticity	High
BBB‐like properties	Incomplete tight junctions, low selectivity	Strong ZO‐1/Claudin‐5 expression, selective transport	High
Disease‐model fidelity	Partial pathological features	Reproduces AD/Stroke/Hypoxia phenotypes robustly	Very High
Drug screening predictivity	Low physiological relevance	High‐throughput and BBB‐relevant drug testing	High

**FIGURE 7:**
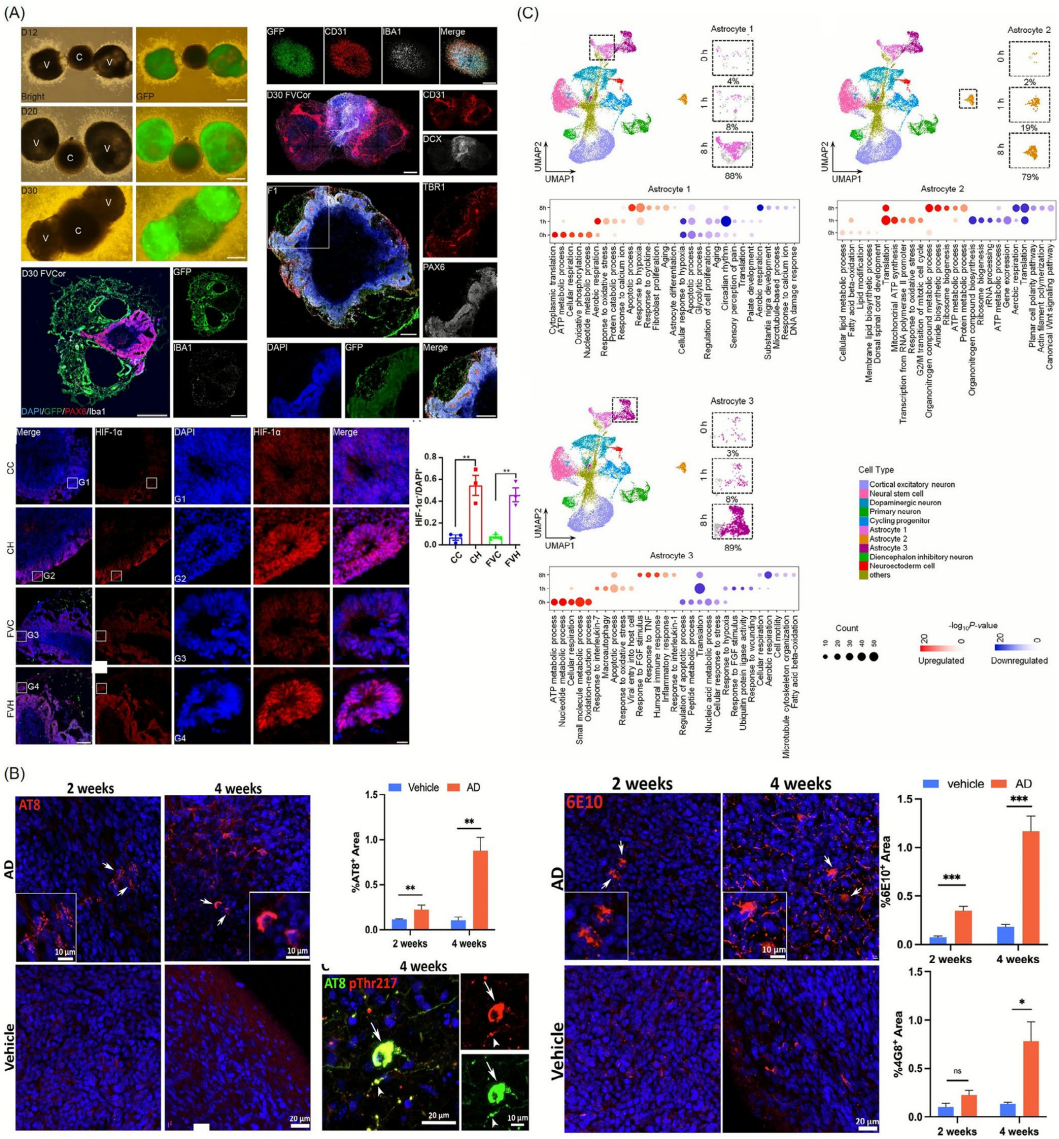
Representative studies of neurological disease modelling. (A) Generation and characterisation of vBOs. vBOs were generated by fusing mesoderm‐derived vascular organoids with endoderm‐derived cerebral organoids, each differentiated from hPSCs. Immunofluorescence analysis showed co‐expression of vascular markers (e.g., CD31, VE‐cadherin) and neuronal markers (e.g., MAP2, TBR2), indicating successful structural integration [112]. (B) Modelling hypoxic–ischemic (HI) injury using vBOs. vBOs were subjected to hypoxic conditions to simulate HI injury. Compared to non‐vascularized organoids, vBOs exhibited stronger transcriptional responses, including downregulation of neurodevelopment‐related genes and enhanced protection of TBR2+ IPs by vascular structures. Single‐cell transcriptomics identified three distinct astrocyte subtypes with specific hypoxia‐induced responses, including one subtype promoting regenerative activity in ischemic conditions. Cortical excitatory neurons showed apoptosis and senescence signatures under hypoxia, confirmed both in vitro and in vivo. This model facilitates mechanistic studies of neuronal injury, inflammation, and vascular dysfunction [113]. (C) Alzheimer's disease modelling and drug testing using vascularized neuroimmune organoids. Vascularized neuroimmune organoids were derived from hPSCs and incorporated neurons, astrocytes, microglia, and vasculature. Upon exposure to brain extracts from patients with sAD, the organoids developed hallmark pathologies, including Aβ plaque deposition, tau hyperphosphorylation, synaptic loss, neuroinflammation, and disrupted neural network activity within 4 weeks [114]. Reproduced with permission.

Recent studies have used vascularized cerebral organoids to model hypoxic–ischemic (HI) injury, leveraging co‐culture or assembloid approaches that incorporate mesoderm‐derived vascular spheroids into cortical organoids to generate perfusable NVU‐like structures [110]. HI injury is a major cause of neonatal encephalopathy. It can begin during pregnancy due to maternal health issues such as placental insufficiency or umbilical cord prolapse and can lead to brain damage caused by oxygen deprivation. Although previous attempts have modelled HI using cerebral organoids, they did not include components such as blood vessels or microglia, which are thought to play critical roles in brain development and injury. In recent work, mesoderm‐derived vascular organoids were fused with endoderm‐derived cerebral organoids to create fused vascularized cerebral organoids [108, 111]. Similar neurovascular assembloid platforms have been shown to support pericyte recruitment, astrocyte–endothelial crosstalk, and dextran‐perfusable vessel formation, underscoring their suitability for modelling ischemia‐induced BBB dysfunction [110]. These organoids exhibited more extensive responses under hypoxic conditions compared to non‐vascularized organoids, with significant reductions in the expression of neurodevelopment‐related genes. Furthermore, the vascular structures appeared to protect T‐box brain protein 2‐positive intermediate progenitor cells (IPs), suggesting that the vasculature contributes to neuroprotection (Figure 7A) [112].

In one study, a hypoxic stroke model was established using brain organoids derived from human induced pluripotent stem cells. Stroke is one of the leading causes of death and disability worldwide, and its pathogenesis and mechanisms are not yet fully understood. This model successfully recapitulated neuronal cell death, inflammatory responses, and vascular dysfunction following ischemic injury. A significant increase in three subtypes of astrocytes was observed, each showing distinct responses to hypoxic conditions. Moreover, a major astrocyte subtype responsible for promoting brain tissue proliferation in ischemic brains was identified. In both in vivo and in vitro conditions, cortical excitatory neurons exhibited signs of apoptosis and senescence after hypoxia. By utilising brain organoids in combination with multi‐omics analyses, the study enabled a precise investigation of the mechanisms underlying hypoxic brain injury (Figure 7B) [113]. These findings are in line with broader surveys of vascularized organoid technologies, which highlight stroke and ischemic encephalopathy as primary use‐cases where controlled vascular remodelling and oxygen gradients are essential readouts [108].

In Alzheimer's disease, vascularized organoids support investigations into amyloid‐beta (Aβ) accumulation, tau hyperphosphorylation, and BBB breakdown in a more physiologically relevant context. Moreover, these organoids can be used to study chronic neurodegenerative processes over extended culture periods, capturing disease progression more faithfully. They also provide a means to explore neuroinflammation and immune cell infiltration, offering insights into diseases like multiple sclerosis and vascular cognitive impairment [114].

A recent study developed vascularized neuroimmune organoids derived from hPSCs. These organoids contain key cell types associated with Alzheimer's disease, including neurons, astrocytes, microglia, and vascular structures. Notably, when brain extracts from patients with sporadic Alzheimer's disease (sAD) were applied to the organoids, hallmark pathological features such as Aβ plaque formation, tau pathology, neuroinflammation, synaptic loss, and impaired neural network function were reproduced within four weeks (Figure 7C). Furthermore, treatment with the FDA‐approved drug lecanemab resulted in reduced Aβ accumulation and increased vascular inflammatory responses, demonstrating a therapeutic effect within the model [114]. These studies collectively position vascularized and neuroimmune brain organoids as next‐generation platforms for modelling sporadic and genetically complex neurodegenerative diseases, enabling simultaneous evaluation of neuronal, glial, and vascular phenotypes in response to patient‐specific insults or therapeutic antibodies.

### 4.2 Drug Permeability and BBB Drug Delivery Test

One of the most promising applications of vBOs is drug screening and BBB permeability testing. These organoids more accurately replicate the selective permeability and tight junction properties of the human BBB compared to traditional 2D or non‐vascularized models, making them ideal for evaluating CNS drug delivery. Recent reviews of vBOs and organoids‐on‐chips similarly emphasise that integrating perfusable vascular networks and neurovascular unit (NVU) components yields more predictive readouts for BBB transport and neurotoxicity than conventional monoculture or Transwell systems [115, 116]. Building on earlier work with multicellular BBB spheroids, several recent studies have optimised 3D co‐culture conditions to generate brain endothelial–pericyte–astrocyte aggregates that reproduce tight junction organisation and transporter expression seen in vivo.

**FIGURE 8:**
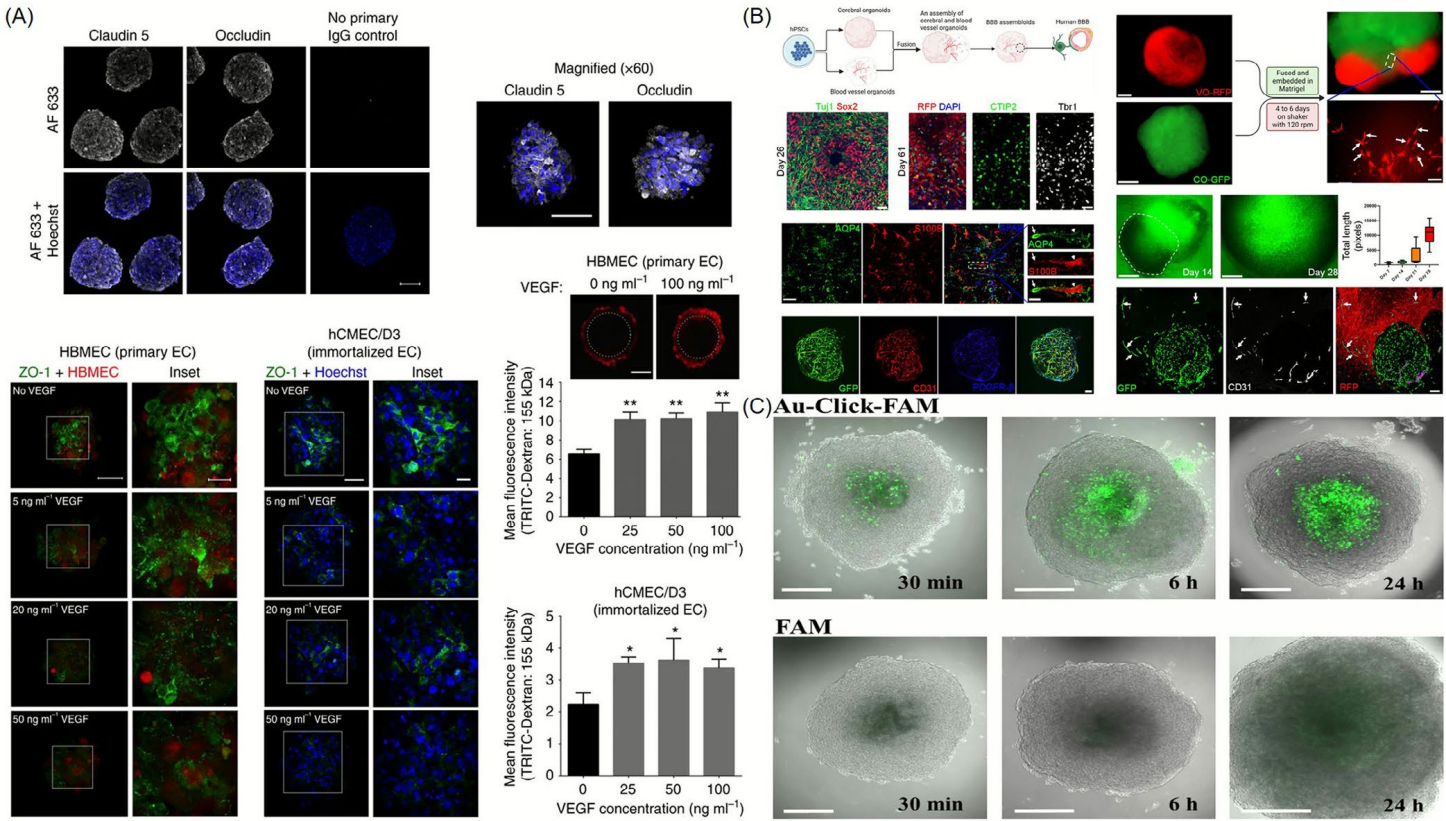
Drug Permeability and BBB Drug Delivery Test. (A) Development and evaluation of BBB spheroids for CNS drug permeability screening. Three‐dimensional blood–brain barrier (BBB) spheroids were formed by co‐culturing human brain ECs, astrocytes, and pericytes. These spheroids expressed tight junction proteins (ZO‐1, claudin‐5), effectively modelling BBB properties. Fluorescent dextran and drug probes were used to evaluate permeability [119]. (B) Vascularized BBB assembloids recapitulate physiological and pathological features of the human blood–brain barrier. Vascularized assembloids were generated by fusing cerebral organoids and vascular organoids derived from hPSCs. Immunostaining confirmed the presence of BBB‐specific markers, and astrocyte and pericyte interactions with endothelial tubes mimicked in vivo architecture [120]. (C) Assessment of nanoparticle‐based drug delivery using vascularized brain spheroids. A six‐cell‐type spheroid model composed of astrocytes, ECs, pericytes, microglia, oligodendrocytes, and neurons was used to evaluate the BBB penetration of ultrasmall Au‐Click‐FAM. Confocal imaging and live‐tracking revealed deep tissue accumulation of nanoparticles within the spheroid core [121]. Reproduced with permission.

In this study, three‐dimensional BBB spheroids were constructed by co‐culturing human‐derived brain ECs, pericytes, and astrocytes [117, 118]. These spheroids expressed tight junction proteins (such as ZO‐1 and claudin‐5) and drug transporters (including P‐gp and GLUT1), effectively mimicking the selective permeability of the BBB. Drug penetration into the spheroids was assessed using fluorescently labelled dextran and candidate drug compounds. In particular, the permeability‐enhancing effects of BBB‐penetrating peptides such as Angiopep‐2 were validated. The spheroids were generated in a 96‐well plate format, demonstrating their potential as a high‐throughput drug screening platform. These vascularized spheroids thus successfully replicated key features of the BBB and were used to evaluate drug permeability across the barrier (Figure 8A) [119]. Integration with microfluidic systems allows real‐time and dynamic testing of compound penetration, efflux activity, and toxicity. This is particularly useful for assessing P‐glycoprotein‐mediated drug efflux, which affects drug retention in the brain. In this study, vascularized BBB assembloids were generated by fusing cerebral organoids and blood vessel organoids derived from hPSCs. Immunofluorescence staining revealed the expression of BBB‐specific markers such as GLUT1, ZO‐1, and claudin‐5 in ECs within the assembloids. These features reflect both the tight junction formation and selective permeability of the human BBB. Additionally, astrocytic end‐feet and pericyte processes were observed to ensheathe endothelial tubes, and functional assessment via TEER confirmed low permeability, mimicking in vivo BBB function. Single‐cell and spatial transcriptomics further demonstrated the presence of major NVU cell types and their spatial organisation. By using assembloids derived from patient iPSCs with Cerebral Cavernous Malformation (CCM) mutations, the study also recapitulated the pathophysiological features of CCMs, including disrupted tight junctions and abnormal vascular morphology. These findings highlight the utility of human BBB assembloids as a robust platform for modelling neurovascular interactions, studying cerebrovascular diseases, and developing CNS‐targeted therapeutics (Figure 8B) [120].

Additionally, vascularized organoids are compatible with high‐throughput screening platforms, and can be used to evaluate nanomedicine strategies, such as liposomes or exosomes, designed to enhance BBB transport and site‐specific drug targeting. In this study, a three‐dimensional brain spheroid was constructed using six major human brain cell types: astrocytes, pericytes, ECs, microglia, oligodendrocytes, and neurons. The spheroid recapitulated key features of the BBB and was used to evaluate the BBB permeability of fluorescently labelled 2‐nm gold nanoparticles (Au‐Click‐FAM). Building on initial observations that ultrasmall nanoparticles can penetrate cell membranes and enter nuclei, the study demonstrated that these nanoparticles could cross the BBB and accumulate in the core of the organoid [118]. This finding indicates that ultrasmall gold nanoparticles can effectively traverse the BBB and be delivered deep into brain tissue, providing a valuable foundation for developing drug delivery systems targeting brain diseases. Additionally, the observed increase in BBB permeability under hypoxic conditions offers useful insights for drug delivery strategies in ischemic disorders such as stroke and supports the utility of this model for assessing the efficacy of nanomedicine‐based therapeutic approaches (Figure 8C) [121].

### 4.3 Cell Therapy and Regenerative Medicine

vBOs hold promise for regenerative medicine and cell therapy, where revascularization is crucial for graft survival and integration. Transplanting pre‐vascularized organoids can repair brain damage from trauma, stroke, or neurodegeneration by rapidly connecting to host vessels and improving oxygen and nutrient delivery. Recent overviews of brain organoid transplantation and vascularized organoid technologies emphasise that pre‐vascularized constructs show superior engraftment, reduced necrosis, and more stable long‐term function compared with non‐vascularized grafts, underscoring vascular integration as a prerequisite for clinically relevant CNS repair [24, 107, 122].

**FIGURE 9:**
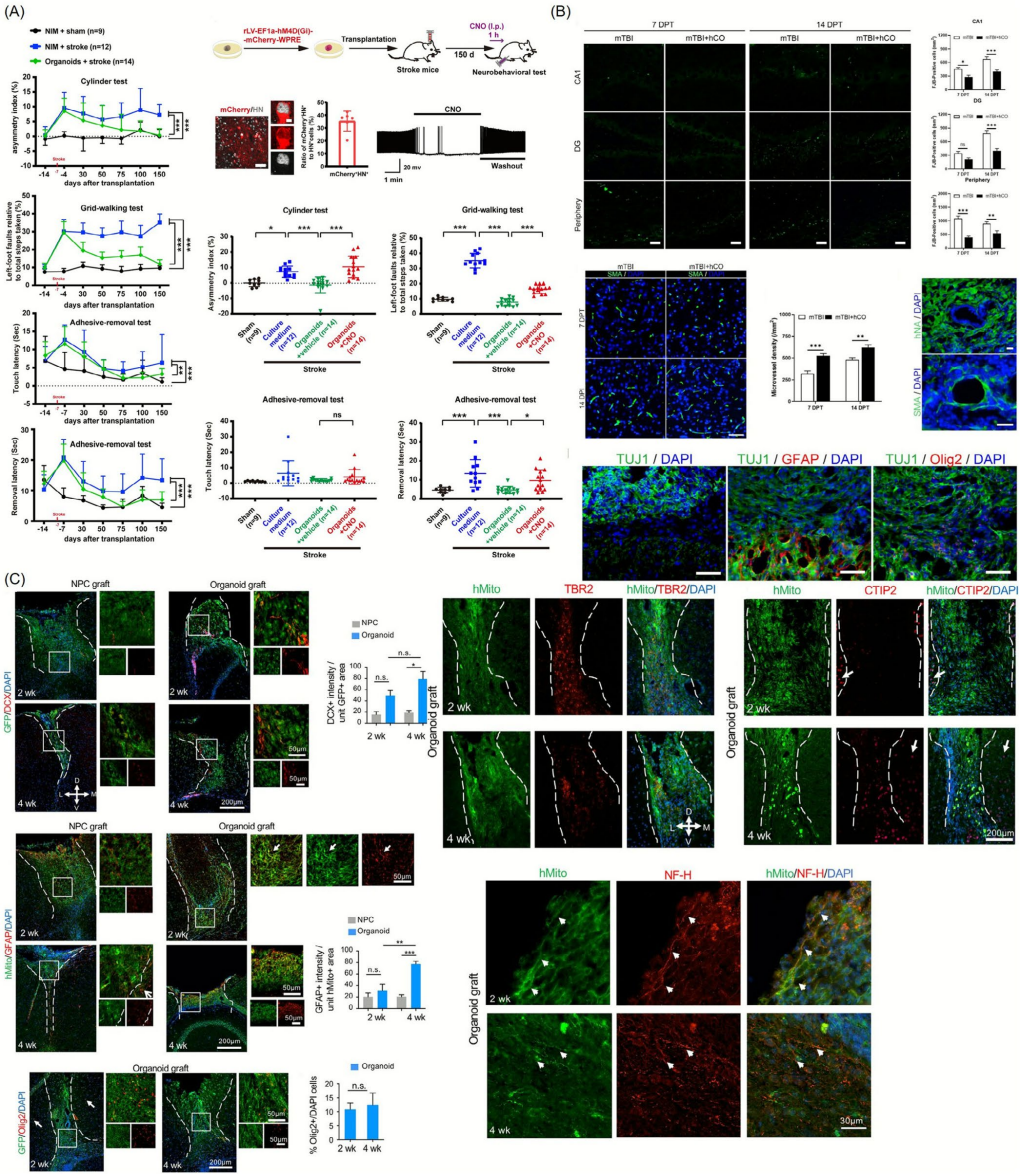
Representative studies that show cell therapy and regenerative medicine using brain organoids. (A) Transplantation of vBOs for neural repair in ischemic and TBI models. hPSC‐derived cerebral organoids were transplanted into the infarct border region of photothrombotic stroke‐injured NOD‐SCID mice. Post‐transplantation analysis revealed organoid survival, neuronal differentiation, and functional recovery in sensorimotor performance [123]. (B) Therapeutic effect of vascularized cerebral organoid transplantation in a TBI mouse model. Cerebral organoids co‐cultured with HUVECs were transplanted into mice with TBI. Immunostaining confirmed vascular integration with host tissue and enhanced survival of grafts. These findings support the importance of pre‐vascularization for graft viability and integration, suggesting that further bioengineering enhancements could increase therapeutic efficacy [124]. (C) Neonatal cortical transplantation of cerebral organoids promotes vascularization and multilineage differentiation. Cerebral organoids derived from hESCs were transplanted into the lesioned cortex of neonatal mice. Host‐derived CD31+ ECs infiltrated the grafts, enabling vascular support. The grafts also expressed NF‐H+ and cortical neuron markers (CTIP2), highlighting their potential for CNS repair and regeneration [47]. Reproduced with permission.

In this study, cerebral organoids derived from hPSCs were transplanted into the junction between the infarct core and the peri‐infarct region of the NOD‐SCID mice that had experienced photothrombotic stroke. The transplanted organoids survived, differentiated into neurons, and contributed to the repair of damaged tissue. Similar stroke‐repair paradigms using human brain organoid grafts have demonstrated that vascular ingrowth into the lesion border is tightly correlated with graft survival and host tissue sparing, highlighting the importance of placing organoids at peri‐infarct interfaces where angiogenic cues are strongest [122]. They also extended axons into distant brain regions, integrating into the host neural circuitry. This integration was associated with the restoration of sensorimotor function (Figure 9A) [123]. Traumatic brain injury (TBI) has recently emerged as a key indication where vBOs may outperform traditional cell suspensions because diffuse axonal damage and chronic hypoperfusion demand both neuronal replacement and rapid vascular support [107, 125]. In this study, vascularized cerebral organoids were transplanted into a mouse model of TBI to evaluate their therapeutic potential. The cerebral organoids were generated from hPSCs and co‐cultured with ECs derived from human umbilical veins to induce the formation of tubular vascular structures within the organoids. Following transplantation, the organoids successfully integrated with the host vasculature and exhibited improved survival and neuronal activity [122]. Immunostaining and behavioural assessments revealed that the transplanted organoids contributed to neural circuit reconstruction and enhanced cognitive functions such as learning and memory. This study highlights the importance of optimising vascularization and maturation of CNS organoids to improve therapeutic outcomes and proposes that incorporating bioengineering approaches such as microfluidic systems to simulate blood flow may further enhance vascular network formation and functional integration in vivo (Figure 9B) [124]. Indeed, recent microfluidic preconditioning studies suggest that exposing vascularized organoids to controlled shear stress before implantation enhances vessel stability and may facilitate faster anastomosis after transplantation [27].

In this study, cerebral organoids derived from human embryonic stem cells (hESCs) were transplanted into the lesioned cortex of neonatal mice to evaluate their potential for neural repair. The 3D‐cultured organoids retained a structured organisation with neural stem cells, neuroblasts, and differentiating neurons. After transplantation, they became robustly vascularized by host CD31‐positive ECs and showed better survival than dissociated neural progenitor cell (NPC) grafts [24]. Immunofluorescence revealed proliferating neural progenitors (Sox2+, Ki67+), neuroblasts (DCX+), astrocytes (GFAP+), and oligodendrocyte lineage cells (Olig2+), indicating multilineage differentiation. The grafts also exhibited nascent axons (NF‐H+) and cortical neuron markers (e.g., CTIP2). These findings demonstrate that cerebral organoids integrate into host tissue, receive vascular support, and undergo neurodifferentiation, supporting their use as a transplantation strategy for CNS injury and as a platform for studying neurodevelopment and regeneration (Figure 9C). Furthermore, autologous iPSC‐derived organoids can reduce immune rejection and serve as a model for studying host–graft immune interactions. Patient‐specific vBOs derived from iPSCs enable personalised disease modelling and drug screening, providing insights into individual disease mechanisms and therapeutic responses [21].

## 5 Current Limits and Future Prospects for Vascularized Brain Organoids

Despite remarkable advances, vBOs still face critical limitations that hinder their full potential as physiologically relevant and clinically applicable models. A major issue is the incomplete recapitulation of the BBB. While some systems exhibit transient tight junctions and selective permeability, these structures often lack full maturity and complexity. Functional assessments, such as TEER measurements and transporter protein profiling (e.g., GLUT1, P‐gp), are inconsistently performed, limiting cross‐model comparisons and validation [29]. Another significant challenge lies in the long‐term stability and functionality of vascular networks. Without continuous perfusion, vascular structures regress over time, compromising nutrient and oxygen exchange. Although in vivo transplantation improves vessel maturation, it limits scalability and raises ethical and technical concerns [60].

Moving forward, it will be important to systematically benchmark the major vascularization strategies in terms of cost‐effectiveness, vascular maturity, and primary application scenarios to guide platform selection for specific therapeutic pipelines. Recent comparative analyses of vBOs indicate that co‐culture and genetic‐induction approaches are relatively inexpensive and amenable to parallelization, making them suitable for medium‐ to high‐throughput disease modelling, whereas microfluidic perfusion systems provide superior BBB fidelity but with higher complexity and lower throughput, positioning them for mechanism‐driven BBB and CNS drug‐delivery studies [108, 116]. By contrast, in vivo transplantation and hybrid strategies achieve the most mature and perfused vasculature, yet their dependence on animal hosts and surgical expertise restricts scalability, suggesting that their strongest near‐term impact will lie in proof of concept regenerative therapies and late‐stage efficacy testing rather than routine screening [24, 122].

Additionally, current vBOs frequently lack key supportive cell types, including astrocytes, microglia, pericytes, and immune cells—critical components for BBB maturation, neurovascular coupling, and modelling inflammatory responses. This absence restricts their applicability in studying neuroinflammation and complex degenerative processes [108]. Most vBO models also resemble foetal brain tissue, with limited ability to capture adult‐like phenotypes. Driving maturation towards more physiologically relevant states requires prolonged culture times and precise biochemical cues, which are difficult to sustain in vitro [126]. Technical reproducibility remains another hurdle, as variability in stem cell differentiation, vascular integration, and 3D culture conditions leads to batch‐to‐batch inconsistencies, reducing their reliability in drug testing and disease modelling [127, 128, 129, 130, 131]. Furthermore, ethical considerations surrounding human‐animal chimeras and patient‐derived implantable constructs demand clearer regulatory frameworks and societal consensus, especially as vBO complexity increases [132]. Future work should therefore prioritise longitudinal assessment of vascular integrity under both perfusion and in vivo conditions, using standardised TEER, permeability, and imaging readouts to quantify vessel regression, leakiness, and BBB phenotype drift over months rather than weeks [103, 117]. Recent organoid‐on‐chip and transplantation studies highlight that shear‐stress–dependent remodelling and pericyte coverage are key determinants of long‐term vascular stability, but also reveal protocol‐specific vulnerabilities such as lumen collapse or barrier breakdown during extended culture, underscoring the need for harmonised maturation criteria across platforms [27]. To specifically address BBB‐associated limitations, emerging nanomedicine platforms—particularly padlock‐designed metal–organic frameworks (MOFs)—have demonstrated considerable promise. These precision‐engineered nanocarriers can trigger an “avalanche effect” in tumour cells, enhancing apoptosis while suppressing metastatic progression [133]. When integrated with vBO systems, MOF‐based drug carriers could enable patient‐specific assessment of drug permeability across the BBB, tumour–vascular interactions, and therapeutic response within physiologically relevant neurovascular microenvironments. Thus, nanomedicine–vBO integration represents a promising complementary strategy to expand the translational utility of vBOs for CNS‐targeted therapeutic evaluation and personalised medicine.

To overcome these challenges, emerging bioengineering approaches are being actively explored. Dynamic microfluidic platforms, 3D bioprinting, and synthetic scaffolds promise better spatial control, hierarchical structuring, and continuous perfusion to stabilise vascular networks. Artificial intelligence (AI) can support the standardisation of culture protocols, while digital twin models may enable predictive simulations of organoid behaviour based on experimental data. Incorporating immune cells, perivascular components, and modelling systemic interactions—such as gut‐brain or neuroimmune axes—could significantly enhance the physiological relevance of vBOs and broaden their applicability to neurovascular disease modelling [115, 134, 135].

Vascularization represents a foundational advancement in organoid technology, enabling more accurate simulation of nutrient exchange, neural maturation, and neurovascular interactions. vBOs surpass conventional organoids in modelling disorders involving the BBB, cerebral perfusion deficits, and neurovascular dysfunction. Beyond improving perfusion, vasculature enhances neural circuit complexity, metabolic regulation, and barrier function, solidifying its importance in disease modelling and therapeutic development. Looking ahead, innovative strategies such as optogenetic vascular control, synthetic vascular patterning, and advanced biofabrication techniques offer opportunities to build precise, dynamic NVUs. Integration with patient‐derived iPSCs and personalised genomic editing will further accelerate precision medicine, while combining vBOs with real‐time biosensor monitoring could advance high‐throughput drug screening platforms. Ultimately, vBOs embody the convergence of stem cell biology, vascular engineering, systems neuroscience, and computational modelling. With continued interdisciplinary innovation, vBOs are poised to become essential platforms for deciphering human brain development, investigating complex neurological diseases, and advancing regenerative therapies and precision medicine.

Looking ahead, translating vBOs into concrete therapeutic development pathways will require aligning specific vascularization strategies with defined clinical questions—for example, using scalable co‐culture or perfusion platforms for BBB‐relevant drug screening in Alzheimer's disease or brain tumours, and reserving highly vascular‐mature in vivo or hybrid constructs for regenerative trials in ischemic stroke, TBI, or leukodystrophies [29, 107]. Emerging precision‐medicine frameworks further envision patient‐derived vBOs being used to stratify responders to biologics, gene therapies, or nanomedicines before expensive in vivo testing, thereby reducing attrition and informing trial design [118].

## 6 Conclusions

1. Vascularization remains one of the most pressing bottlenecks in brain organoid research, fundamentally limiting organoid size, neuronal maturation, and physiological fidelity. Without adequate blood vessel formation, diffusion limits impair oxygen and nutrient delivery, leading to hypoxia and necrosis in larger constructs. Addressing this barrier is essential to unlock the full potential of brain organoids in disease modelling and translational medicine.
2. Recent bioengineering approaches—such as endothelial cell co‐culture, transcription factor–mediated vascular induction, microfluidic perfusion, 3D bioprinting, and hybrid strategies—have collectively advanced the structural and functional integration of vascular networks. These methods not only improve the survival and maturation of neuronal populations but also facilitate the establishment of BBB‐like properties within the organoid microenvironment.
3. Despite these advancements, current vBO systems face substantial limitations, including incomplete BBB recapitulation, instability of vascular networks over long culture periods, and significant batch‐to‐batch variability. These constraints hinder reproducibility, scalability, and widespread adoption of vBO technology across laboratories.
4. The integration of next‐generation tools—dynamic perfusion bioreactors, biomimetic synthetic scaffolds, and AI–driven optimization of culture parameters—offers a promising route to overcoming current hurdles. Such technologies can enable continuous nutrient and oxygen delivery, refine spatial patterning of vasculature, and enhance experimental reproducibility.
5. Translational applications of vBOs are emerging rapidly, with potential in modelling complex neurovascular disorders, assessing BBB permeability for CNS drug development, and serving as platforms for regenerative medicine. However, successful clinical translation will require standardised protocols, regulatory alignment, and multi‐sector collaboration involving academia, industry, and healthcare systems.
6. The future of vBO research lies in the sustained convergence of stem cell biology, biomaterials engineering, and organ‐on‐a‐chip technologies. Cross‐disciplinary partnerships will be vital to develop physiologically accurate, scalable, and customizable brain organoid platforms that faithfully model human neurovascular biology and support breakthroughs in neuroscience and medicine.

## Author Contributions

Conceptualization, Y.S., H.J., S.J. and S.S.L.; Investigation, Y.S., S.J. and S.S.L.; Writing – Original Draft, Y.S., S.J.and S.S.L.; Writing – Review and Editing, Y.S., H.J., S.J., I.K. and S.S.L.; Visualization, Y.S., H.J., S.J. and I.K; Supervision, I.K. and S.S.L.; Funding Acquisition, S.S.L.

## Funding

This work was supported by the National Research Foundation of Korea, RS‐2024‐00415982, RS‐2025‐23525049, RS‐2025‐02633264, S‐2024‐00450843, RS‐2025‐25460008; Korea Institute for Advancement of Technology, P241200036; Dongguk University Research Fund, S‐2024‐G0001‐00024.

## Conflicts of Interest

The authors declare no conflicts of interest.

## Data Availability

The data that support the findings of this study are available from the corresponding author upon reasonable request.
